# Rules of engagement: perspectives on stakeholder engagement for genomic biobanking research in South Africa

**DOI:** 10.1186/s12910-018-0252-y

**Published:** 2018-02-27

**Authors:** Ciara Staunton, Paulina Tindana, Melany Hendricks, Keymanthri Moodley

**Affiliations:** 1 Department of Medicine, Centre for Medical Ethics and Law, Faculty of Health Sciences, Stellenbosch University, Stellenbosch, South Africa; 2 Navrongo Health Research Centre, Ghana Health Service, Navrongo, Ghana

**Keywords:** Community engagement, Ethics, Genomic biobanking research, H3Africa

## Abstract

**Background:**

Genomic biobanking research is undergoing exponential growth in Africa raising a host of legal, ethical and social issues. Given the scientific complexity associated with genomics, there is a growing recognition globally of the importance of science translation and community engagement (CE) for this type of research, as it creates the potential to build relationships, increase trust, improve consent processes and empower local communities. Despite this level of recognition, there is a lack of empirical evidence of the practise and processes for effective CE in genomic biobanking in Africa.

**Methods:**

To begin to address this vacuum, 17 in-depth face to face interviews were conducted with South African experts in genomic biobanking research and CE to provide insight into the process, benefits and challenges of CE in South Africa. Emerging themes were analysed using a contextualised thematic approach.

**Results:**

Several themes emerged concerning the conduct of CE in genomic biobanking research in Africa. Although the literature tends to focus on the local community in CE, respondents in this study described three different layers of stakeholder engagement: community level, peer level and high level. Community level engagement includes potential participants, community advisory boards (CAB) and field workers; peer level engagement includes researchers, biobankers and scientists, while high level engagement includes government officials, funders and policy makers. Although education of each stakeholder layer is important, education of the community layer can be most challenging, due to the complexity of the research and educational levels of stakeholders in this layer.

**Conclusion:**

CE is time-consuming and often requires an interdisciplinary research team approach. However careful planning of the engagement strategy, including an understanding of the differing layers of stakeholder engagement, and the specific educational needs at each layer, can help in the development of a relationship based on trust between the research team and various stakeholder groups. Since the community layer often comprises vulnerable populations in low and middle income countries (LMICs), co-development of innovative educational tools on genomic biobanking is essential. CE is clearly a component of a broader process best described as stakeholder engagement.

## Background

Genomic biobanking and associated research create unique scientific and ethical challenges globally. Research in genomics is undergoing exponential growth in Africa as a result of international collaborative projects such as HapMap, MalariaGEN, H3Africa, B3Africa and other local collaborations. While South Africa does not have population based biobanks currently, there are several disease specific specimen collections in the country. Scientists in South Africa have become a part of these international initiatives, and have complemented efforts by the Department of Health and the National Health Laboratory Service to develop and promote genomic biobanking in the region [1]. Although welcomed, the research raises a host of legal, ethical and social issues for the continent, including challenges with the traditional understanding of consent information, questions of benefit-sharing, ownership, fears of exploitation, discrimination and stigmatisation [2, 3]. These add to the well-documented challenges associated with conducting research in vulnerable populations in LMICs [4, 5]. Many of the difficulties genomic and biobanking researchers in South Africa face have been experienced in other High Income Countries (HIC), but genomic and biobanking research present particular ethical challenges in South Africa. The country’s history of exploitation has impacted negatively on trust in medical research [6] and attempts at data and sample mining persist [7]. Empirical studies have suggested that biological samples have a cultural significance that must be respected and can affect the use and reuse of samples [6]. Furthermore, the complexity of genomic biobanking research coupled with low literacy rates in many communities, and the lack of vocabulary for many terms in indigenous African languages can also challenge understanding during consent processes [8].

Research on Human Immunodeficiency Virus (HIV) infections and genetics and genomics raise similar ethical challenges. Both disease entities share the phenomenon of scientific complexity, transmissibility and the potential for stigmatisation for different reasons. It is therefore unsurprising that HIV research in Africa has similarly raised considerable ethical and social issues over the past three decades, and CE has been demonstrated as a key way to address some of these issues [9–12]. CE in health research typically refers to the efforts of researchers “to develop partnerships with local stakeholders and to involve them in assessing local health problems, determining the value of research, planning, conducting and overseeing research, and integrating research into the health care system” [13]. It assumes that interventions that are community based and involve all major stakeholders are often more effective, sustainable, and more likely to reflect the health needs of the communities while promoting capacity development [14]. There has been a growing body of literature on the importance CE for the ethical conduct of research [2, 11, 15, 16]. Although the goals will vary according to the research and its setting, building relationships, increasing trust, improving the informed consent process and empowering local communities is critical to the success of all research projects [12, 17, 18]. Essential to this process is an understanding of the research [19].

Despite the perceived importance of CE in promoting the ethical conduct of research, challenges in its implementation and establishing its impact remain. Defining ‘community’ is challenging and can depend on the particular social, cultural and geographical context in which the research takes place. The conception of CE can similarly vary and community involvement, consultation, participation or partnership have all been considered to be CE [2]. Questions also persist as to what constitutes meaningful engagement [20]. The difficulty in defining CE additionally makes the effective evaluation of CE challenging. These challenges are particularly pertinent as there is a lack of guidance as well as empirical evidence of the practise and processes for effective CE in genomic biobanking in Africa. A 2015 review by Tindana et al. revealed that out of the 38 published articles on CE in sub-Saharan Africa, 34 related to biomedical research with only 4 specific to genetic and genomic research in Africa. The H3Africa Community Engagement Guidelines remain the only CE guidelines for genomic biobanking research that is specific to the African continent [21].

Within South Africa, previous studies have explored research participant perceptions around the use of biological samples in research [3], the views of researchers regarding perceived ethical issues in genomic biobanking [6] and experiences with a researcher-driven, population-specific CAB [18]. CE was identified as an important contributor to building trust in genomic biobanking research [6], but there has been a paucity of empirical work in CE and genomic biobanking research in South Africa. We therefore conducted interviews with experts in CE as well as key stakeholders in genomic biobanking research in South Africa, to explore some of the pertinent issues concerning CE in the country. This paper reports on these findings and is the first study to focus exclusively on CE in genomic biobanking research in South Africa. This research will guide future phases of work involving empirical investigation into similar issues with patients and community advisory board members (CAB). The findings of this work will inform the development of a CE strategy for a biobank in Cape Town, and may inform the development of similar guidelines for urban biobanks across Africa.

## Methods

This was a qualitative research project involving face to face in-depth interviews with expert stakeholders. Using purposive sampling, we selected respondents with expertise in genomics and biobanking research and/or CE to provide insight into the process, benefits and challenges of CE in South Africa (see Table 1). Within these parameters, the research team used their collective experience and network to identify respondents. Respondents were identified through some of the research teams’ (CS, PT, KM) membership of various H3Africa Working Groups and the experience and involvement of the research team with CE for research in sub-Saharan Africa (PT). We also drew on the teams’ experience (CS, MH, KM) and network developed with researchers in South Africa who were not part of the H3Africa Consortium, but had experience in CE and/or genomic biobanking research in South Africa. Respondents were confined to those who resided in the Western Cape, or who were available to take part in an interview in Cape Town between March and June 2016. Respondents included medical scientists, biobank staff and CE practitioners. Although all respondents were based in South Africa, many are involved in multisite projects with collaborators across the continent, thus some of the results may have implications for CE throughout Africa.

**Table 1:** Breakdown of respondents

Researchers (Genomic and biobank researchers)	10
CE experts	3
Lawyer	1
Study nurse	1
Genetic counsellor	1
Genetic counsellor & member of patient advocacy group	1

The 17 in-depth interviews took place in Cape Town and lasted approximately 50 min each. After outlining the project and the purpose for the interview, written informed consent for participation and recording of the interview was obtained. Interviews explored participants’ experience with CE in the context of genomics biobanking research. Questions focused on respondents’ perspectives on the goals and purpose of CE, how CE should be structured as well as challenges with CE generally. Respondents who had experience with genomic biobanking research were asked to reflect on these issues in the context of that research. As part of this project, interviews with 10 CAB members and 30 research participants were ongoing at the time of submitting this paper. These 40 interviews were conducted independently of the interviews reported in this paper, and are examining CAB and research participants experiences and perspectives of CE in South Africa. The findings of those interviews will be published at a later date and will be compared with the findings in this paper.

All interviews were conducted in English, recorded and transcribed verbatim. A codebook was developed by the interviewer (CS) and discussed with two team members (PT, MH). Emerging themes were discussed with the principal investigator (KM) and the team (CS, PT, MH). Data analysis was facilitated by using the software Nvivo. A contextualised thematic approach was used to interpret the results. This study was approved by the Research Ethics Committees at Stellenbosch University N14/02/010 and the University of Cape Town 084/2016.

## Results

All respondents noted the importance of CE generally, its importance in research, and that CE should be included as an integral part of the research process for the ethical conduct of research. Trust, CE as a means to empowerment, reciprocity and feedback to a community, and the importance of evaluating CE were all highlighted as being of significance. In discussing these elements, respondents touched on the value of these issues as they relate to genomic biobanking research. In particular, trust was identified as being significant in this context, as key concepts of the research itself and other issues, such as broad consent, may not be understood.

*I don’t think that our community fully understands the whole issue of what broad consent is…but I think usually the scenarios that work is an element of trust. I will get a 90% hit for a sample for bio banking because of the nature in which I have engaged with people.* (Researcher, 01)

For the purposes of this paper, we present the key themes that emerged from the interviews as they specifically relate to genomic biobanking research in South Africa.

### Layers of engagement

Although the questions sought to explore CE, respondents discussed the different layers of engagement that may be necessary prior to, during and after the research. They reflected upon the differing layers, the type of engagement that is necessary and the justification for engagement with each layer.

Although engagement with ‘the community’ was most discussed, the three different layers of engagement described by respondent were:

- High level: policy makers, institutions and funders
- Peer level: scientists, medical doctors, biobankers, nurses and field workers
- Community level: community members, patients, research participants, support groups, field workers and CABs

These differing layers were explicitly described by one respondent who had considerable international experience in multi-site projects, but were echoed by other respondents as well. The attention given to each layer varied amongst respondents. Researchers discussed all layers, but gave most attention to the community level and high level, while CE experts tended to focus on the peer and community level of engagement.

*So there are three levels of CE. There's high level, there's peer level and then there’s community and public.* (Researcher, 04)

#### High level engagement

High level engagement was considered to include those stakeholders who are involved in decision making on an institutional, national or international level. This could include government officials, policy makers, funders or a hospital director/superintendent. In discussing the manner and purpose of engagement, it was felt that this will vary according within each level, and this layer could perhaps be subdivided between those who are developing policy and financially supporting the research, and those whose approval or permission is necessary prior to the commencement of the research.

For policy makers and funders, high level engagement may be necessary to change policy, motivate for increased funding or obtain the necessary approvals and permissions for the research. There was recognition that genomic biobanking research is expensive and it may take time before there is a clinical application from the research. However, considerable financial investment is required to ensure its sustainability, and economic arguments demonstrating the benefits of genomic biobanking research may be needed for these discussions.

*And of course the political leaders are critical, because every single thing you do at the end you need to have it implemented, especially if you are doing health research.* (Researcher, 10)

*High level policy makers have very short attention spans and you need to wrap their focus and drive home your messaging in minutes. If you don’t create an economic argument they are not interested so just to try to convince them that they need to spend money on R and D is not going to fly. You need to explain to them why or what the benefits are in terms of loss of revenue or overspending because of lack of a proper strategic plan.* (Researcher, 04)

#### Peer level engagement

At some level, all respondents discussed the need for peer engagement. The concept of a peer generally referred to someone with a medical background who would be involved in the research in some way. Once again, there was a split within this layer, between those leading the research and those who directly engaged with the patients.

In reflecting upon peer engagement, respondents considered that it may be necessary for two purposes: to develop collaborations for research and to educate. The purpose of education can be twofold. First, respondents spoke about the importance of demonstrating to scientists in particular, the importance and need for CE and the benefit of a better informed community. Respondents noted that effective monitoring and evaluation of CE can help justify CE, as this will provide evidence of its effectiveness. They also need to be engaged and educated about how to conduct CE. The development of these collaborations could occur at scientific meetings and conferences where CE could be included as part of the programme.

Most respondents emphasised the importance of engaging and educating frontline staff such as researchers, recruiters and field workers who will be interacting with participants. They recommended that doctors and other medical professionals need to be made aware of the importance of genomic biobanking research so that they are willing to refer patients. Although frontline staff may have a medical background, their knowledge about genomics and biobanking research cannot be assumed. In particular, respondents opined that field workers may require in-depth training to ensure that they are all conveying the same message.

*And then we also need to support the efforts of the individual researchers, because a lot of them may not have the resources or the expertise or the interest to do effective CE.* (Researcher, 04)

*We assume that the staff knows what they are doing, even some of the nurses that are recruiting people in the genomic biobanking projects or in biomedical research or genomic research, we assume that the nurses understand the genomics. But no one has taught them, so why should we assume?* (Researcher, 08)

#### Community level engagement

Identification of the community layer was less explicit, and difficulty in defining the community was discussed. This is partly due to the difficulty in defining this group, but it is also dependent on whether it is a population based study or disease specific. It did, however, extend to including patients, members of support groups, a community advisory board (CAB), field workers, patients with a specific genetic condition and the general public. For some, field workers could also occupy this layer as they may be drawn from the community they are working in, but it was acknowledged that they may have a conflicted role as both members of the community and part of the research team. The purpose of engagement with this layer is to educate and build trust, but similar to the high level layer, the length of time it can take to achieve results, was perceived to be challenging.

*It would depend a lot on the type of research that you want to do. If it is research that involves families it’s very, very different. My engagement would be much more different than research that engages individual people…If you have to do monogenic research for gene hunting that means you will have to deal with family. Your engagement will be almost on a family basis and individual family approach* (Researcher, 10)

*I think that if you speak to the recruiters that they’ll say it’s a very conflicted role because on the one hand they are X people who are members of this target community. And so, they are gate keepers and they also feel a sense of advocacy for the rights of the participants.* (CE expert, 02)

### Education of stakeholders in the community layer

The importance of educating each layer was discussed, but the purpose of education of each layer differed. By far the most attention was devoted to education of the community layer. The importance in educating this layer was deemed to be critical but there was the feeling that educating about genomic biobanking research is particularly challenging and that considerable time to explain genomic concepts to the patient is necessary. Challenges related to the complexity of the topic, the difficulty in making the information accessible, and the low education levels of many who are likely to be part of this research in South Africa. However, respondents commented that while there are difficulties with educating the community about genomics and biobanking, it is not impossible and efforts should be made to make the information accessible.

*So access to that knowledge is difficult but it doesn't mean that it is impossible. It just means that we need to find a way to make it accessible to the people.* (Researcher, 10)

Education was also perceived to be crucial to alleviate concerns of the community about the research and de-stigmatise feelings of blame about their children inheriting any genetic disorders.

*It's about self and it's about the future. It is also about going back and it's about guilt and blame, about where it comes from and why me? And so we have to go back to be able to go forward and often we get a stumbling block going back, because then there is blame. And so we have to reinforce that and you know go through the process that your parents didn't know that they were…carrying this condition* (Researcher, 09)

Education was also deemed essential to address concerns *about* exploitation and to *prevent* exploitation. Informing the community about the African ownership of the research and that the samples will remain in Africa was considered essential to allay fears of exploitation. Respondents did touch on concerns about the over-research of participants in Cape Town and the South African public health sector generally. One respondent raised particular concerns about the attempt of the health insurer, Discovery Health, to obtain samples from patients in return for reduced cost genetic testing as patients may not understand the implications of this test.

*You would be surprised at what community members bring to the table when it comes to research…as you might know some of the research that has been done where samples have had to be sent away… But a biobank that is based in Africa that has stakeholders from Africa and who decides over issues that happen in Africa is different.* (CE expert, 03)

*Genetic information is very sensitive. It's very powerful. I mean it can be used to discriminate against you* [Discovery] *are making it available to the general public and they don't know a lot about genetics and what it actually entails.* (Genetic counsellor, 01)

Finally, the significance of educational tools in overcoming this difficulty was highlighted. Such educational tools were necessary at all layers of engagement, not only the community level. There was the sense that additional tools were needed to begin to unpack this complex subject matter. Social media, newsletters, posters, pamphlets, videos, science exhibitions, workshops/online courses and generic or disease specific awareness days were all mentioned as possible educational tools. These tools, in addition to verbal discussions, were felt to be important as information delivered orally should be backed up with some other intervention. Attention was also given to the words and metaphors that can help stakeholders in the community layer better understand the research. Examples provided included soap operas to explain paternity, cookies and recipes to explain genetics, and toy soldiers to explain HIV.

### Respecting cultural norms and practices

For the peer group, respondents noted that education is essential so that peers respect cultural norms and practices common in some South African communities. Failure to do so may result in delays to the research. For example, access may only be obtained through the chief or elders in more rural settings and there may be a process to follow before informed consent can be obtained. It was noted that many of the communities that researchers work within are patriarchal and the consent of a husband or father may be necessary prior to the consent of a woman. Similarly community consent or support or discussions with family may be necessary prior to individual consent. In such settings this tradition must be respected and followed.

*Like I know for our Xhosa patients for instance…they need to go to the community leaders or to their elders…if you're trying to get someone's consent for something like this they might say to you “Well, I'm not the decision maker” so that could also be a problem and I think that you should think about this when you do the recruitment process.* (Genetic Counsellor, 01)

Turning specifically to genomic biobanking research, the cultural significance of the samples and understanding the cultural narrative around disease in many South African communities were particularly emphasised.

In discussing the samples, respondents noted the cultural beliefs and concerns on the withdrawal of blood, the taking of samples and the storage of hair. Many South African traditional communities believe that they will linger in this life if their sample remains in a fridge but there are also fears held that their blood will be used for “muti” or witchcraft. They also believe that donating samples may be offensive to their ancestors in some way. These beliefs must be understood and respected.

*They contacted the family back in the rural area who agreed they’ll come and they will give samples; and then they* [the researchers] *never really travelled there to get them. And they were told “No way, you know one of the elders has pointed out that this is totally disrespectful to the ancestors and we don’t want to give samples for this.” And they didn’t, they wouldn’t.* (Researcher, 07)

*Then you sit with…different traditions. Some people believe, especially in the various cultures that you know if a piece of the body is a sample in the fridge… they can’t move on.* (Researcher, 03)

Respondents noted that perceptions of disease and illness in some communities may differ from a biomedical understanding. In explanations of disease, the team must be sensitive to the cultural understanding of the disease; both the biomedical and cultural explanations must be respected.

Similarly explanations of inheritance of disease may be considered disrespectful to the ancestors. One respondent reported that a family refused to give samples as the elders believed it to be disrespectful to the ancestors. For others, they may struggle to accept certain explanations due to their belief in God and there may be a certain discomfort around discussions about sex and sexuality. Researchers must be prepared for these concerns and queries and be in a position to address them.

*And then we engage in conversations about how the genetic story intersects with their own narrative and I think it’s been a win/win because that way people get to question and ask questions about what the genetics is and what their own oral history is about.* (Researcher, 01)

*Also respecting how a particular community or members within the community will explain and understand illness and health seeking behaviour around that explanation. And how that aligns with maybe how the project is talking about that particular illness and respecting both those different explanations* (CE expert, 02)

## Discussion

CE in any context is challenging, and the development and implementation of a CE strategy requires time, patience and resources as well as considerable interpersonal skills and flexibility [11]. South Africa has considerable experience in CE for HIV research, and the lessons learned in that context should be drawn upon for genomic biobanking research. However, the complexity of genomic biobanking research, the capacity of those involved in engagement, and the cultural and societal concerns that are drawn out by the research, pose additional challenges to CE.

CE is recognised as intrinsically respecting and protecting communities, but engagement for this research must go beyond the community layer, as engagement of each stakeholder layer is important to ensure the success of genomic biobanking research in Africa. Most published work on CE for genomic biobanking research often tends to emphasise CE at the level of the target community or population hosting the project [18], but our research has highlighted the need to take other layers of engagement seriously, such as peer engagement with other scientists and high level engagement with policy makers which can influence the uptake of best practices. Fundamental to CE is recognition of its importance in the ethical conduct of research. This must be understood and recognised by those in the peer and high level layer to ensure that adequate resources are dedicated to CE. Engagement with these layers can ensure that CE becomes an integral part of good governance for genomic biobanking research. Thus our focus in genomic research in South Africa must go beyond CE to encompass stakeholder engagement.

Increasingly, there is recognition of the importance of stakeholder engagement in HICs in the field of genomic biobanking research [21–24]. It has been referred to as “the process of meaningful involvement of those who are engaged in making decisions about programs” [21]. For each layer to be involved, there must be an understanding of the research and education of the different stakeholder groups is therefore essential. To an extent, the H3Africa Consortium is already engaged in this process, but gaps nevertheless remain.

H3Africa Consortium meetings are generally held twice a year with principal investigators (PIs), co-investigators, members of the various Working Groups as well as funders in attendance. Successes have led to the decision to fund H3Africa for a further 5 years and the requirement that 5% of all grants include a CE plan. However, national funders generally are not in attendance and there is no formal process for researchers to engage with such bodies in South Africa. Sustainability of genomic biobanking research post-H3Africa has been raised as a concern [25], thus there is a need for genomic biobanking researchers in South Africa to start this process of engagement to ensure its continued development and success.

Equally, the H3Africa Ethics and Regulatory Issues Working Group has developed an *Ethics and Governance Framework for Best Practice in Genomic Research and Biobanking in Africa*. This framework was developed after engagement with some REC members, members of the H3Africa Consortium and regulators from specific African countries [2]. To a certain extent, this framework can help support individual RECs, as well as researchers in South Africa to ensure the ethical conduct of genomic biobanking research. However, broad consent can never be truly informed as possible risks cannot be known and thus remains contentious [26–28]. Gaps in the regulation of genomic biobanking research in South Africa have been identified [7, 29], and this framework can help address some of these shortcomings, including informing the development of specific guidelines on genomic biobanking at institutional and national levels.

Similarly, the H3Africa Community Engagement Working Group is involved in engaging with the Consortium generally, and is working on individual projects looking at the importance of CE and providing advice in the development of CE plans [30]. Looking beyond the Consortium, there is a need to focus on stakeholder engagement rather than CE only.

The three layers of engagement described by respondents in this study, however, are not rigid, but will vary according to the research context. Nurses and other medical personnel were identified as potentially engaging with and recruiting participants, but they were seen as separate from field workers. With the exception of one respondent who was a CE expert, field workers were perceived by respondents to be a part of the community layer. As they are part of the research team, field workers may be perceived differently by community members [31]. This demonstrates that it is not possible to definitely group individuals. Individuals can occupy different layers depending on the particular conceptualisation of ‘community’ [10] and, although they may be a community member educated in the research, they can perhaps continue to represent their community. However questions may arise over whether the community perceives these field workers as representing them and assumptions cannot be made about the layer that field workers may represent. Whatever the layer, fieldworkers have an important role in the informed consent process, helping participants to conceptualise the research. It is therefore essential that they receive adequate training [18, 31, 32].

Equally, a better understanding of the role and the layer that fieldworkers occupy will also be important in future evaluation. If field workers are part of the peer layer, their role will be in ensuring that the community level is engaged and understands the research. They will have an active role in CE and bolstering the consent process. On the other hand, field workers occupying the community layer will have a more active role in representing the community and voicing any concerns associated with the research. In reality such roles for field workers are not mutually exclusive and perhaps cannot be delineated, but the community’s perception of their role must be better understood.

For each layer, education is important and implicit in this is the need for co-learning [33], particularly between the peer and community layer. Across both layers, problems understanding genomic biobanking research persist and suggestions for capacity development of the peer layer for genomic biobanking research [25] and research generally have been discussed elsewhere [34]. Updating undergraduate medical curricula to include modules on genomics and biobanking is essential. Likewise the development of educational courses for medical doctors, REC members and scholars of law and ethics is important.

The challenges surrounding the complexity of genomic and biobanking research, language issues, coupled with the cultural sensitives in some communities, was seen as the greatest challenge to genomic and biobanking research in Africa. Challenges in engaging communities where there are low levels of literacy means there are often low levels of awareness and understanding of research and this can lead to the diagnostic misconception in the context of genomic biobanking research [35].

There have been calls for simple and accessible language in conjunction with visual aids to explain genomic research [18] and a growing body of literature has demonstrated the effectiveness of such tools in South Africa and other LMICs [36, 37]. It is clear from our findings that there are on-going efforts to simplify the language in which information is presented as well as develop appropriate educational tools.

In an analysis of H3Africa consent forms, it was found that there were broadly five strategies employed to explain genetics and genomics that focused on heredity, heredity and health, genes and disease causation, disease susceptibility and progression and phenotype or a combination of these strategies [38]. However, consent forms in indigenous African languages were not included in that study, and it is unclear whether the lack of vocabulary for scientific terms such as “gene”, “DNA” or “biobank” in many African languages has been addressed.

As part of our project, we developed a video on biobanking research and educational pamphlets that further discuss medical research, genetic and genomic research, and biobanks. This video has been made publicly available on You Tube [39] as well as in the H3Africa Consent Guideline. Both the pamphlets and the video have been disseminated at conferences and workshops, to researchers and REC members, and made available to individual clinics and research participants. RECs are disseminating the videolink and pamphlets to researchers as most studies today have a separate consent process for the collection and storage of samples for genomics research in the future. It is essential to not only disseminate to research participants, but also to stakeholders in the peer layer, given that they educate stakeholders at the community level. The recently revised H3Africa Community engagement guidelines provide examples of other educational tools that have been used within the H3Africa consortium including comic books, pictographs, theatrical engagement, and social media to engage their target communities.

Finally, the cultural issues raised by genomic biobanking research, and the need to respect local narratives and customs, are similar to particular challenges with this research discussed elsewhere [3, 6]. While attempts to develop research capacity for genomic biobanking research are indeed to be welcomed, concerns about the potential exploitative nature of this research remain [7, 25]. Given South Africa’s history of exploitation, this is perhaps a pertinent issue for each layer. Equitable research collaborations is one step to address such fears [25], but a more robust regulatory framework can also address this issue.

### Limitations

A limitation with this publication is that it focused on the perceptions of researchers, genetic counsellors and CE experts only. Further empirical work on the views of those who have or are likely to be involved in genomic and biobanking research such as research participants, fieldworkers and research ethics committees is necessary. As part of this project, the views of research participants and CAB members are being elicited and the views in this paper will be compared with the views that will emerge from research participants and CAB interviews.

A further limitation lies in the sampling method adopted. Respondents were generally drawn from the peer level and quite senior. They were also researchers who were PIs of research projects that were actively engaged in CE. The views of these respondents are biased in favour of CE and may not be reflective of all researchers engaged with genomic and biobanking research. Due to the network of the research team, the respondents were mainly drawn from the H3Africa Consortium. However, most respondents were involved or are currently involved in research projects outside of the H3Africa network, thus the findings are likely to be representative of views beyond the H3Africa network. Finally, many of the respondents drew on their experiences that involved work in other African countries and these findings may have implications for research beyond South Africa, however further work in differing contexts across sub-Saharan Africa is necessary.

## Conclusion

There are many challenges in CE in genomic biobanking research in LMICs due to the complexity of scientific and ethical concerns coupled with educationally disadvantaged participants with low levels of scientific knowledge and language and communication challenges. The impact of culture, educational approaches and community empowerment can all have an important bearing on how to interact with a community to begin a CE process. Engagement must however extend beyond stakeholders in this community layer and encompass high level and peer engagement as well. Many medically qualified health workers have limited knowledge on genomics and biobanking. The study has shown that CE is merely a component of a broader concept, namely, stakeholder engagement.

## Abbreviations

CAB: Community advisory board
CE: Community engagement
HIV: Human Immunodeficiency Virus
M&E: Monitoring and evaluation
REC: Research ethics committee
